# Impact of Nano- and Micro-Sized Chromium(III) Particles on Cytotoxicity and Gene Expression Profiles Related to Genomic Stability in Human Keratinocytes and Alveolar Epithelial Cells

**DOI:** 10.3390/nano12081294

**Published:** 2022-04-11

**Authors:** Paul Schumacher, Franziska Fischer, Joachim Sann, Dirk Walter, Andrea Hartwig

**Affiliations:** 1 Department of Food Chemistry and Toxicology, Institute of Applied Biosciences (IAB), Karlsruhe Institute of Technology (KIT), Adenauerring 20a, 76131 Karlsruhe, Germany; paul.schumacher@kit.edu (P.S.); franziska.fischer@kit.edu (F.F.); 2 Institute of Physical Chemistry, Justus-Liebig-University Giessen, Heinrich-Buff-Ring 17, 35392 Giessen, Germany; joachim.sann@phys.chemie.uni-giessen.de; 3 Center for Materials Research (LaMa/ZfM), Justus-Liebig-University Giessen, Heinrich-Buff-Ring 16, 35392 Giessen, Germany; 4 Laboratories of Chemistry and Physics, Institute of Occupational and Social Medicine, Justus-Liebig-University Giessen, Aulweg 129, 35392 Giessen, Germany; dirk.walter@arbmed.med.uni-giessen.de; 5 Institute of Inorganic and Analytical Chemistry, Justus-Liebig-University Giessen, Heinrich-Buff-Ring 17, 35392 Giessen, Germany

**Keywords:** Cr_2_O_3_ particles, Cr(VI) release, cytotoxicity, gene expression profiles, DNA damage signaling, DNA repair proteins, oxidative stress, cell death pathways

## Abstract

Exposure to Cr(VI) compounds has been consistently associated with genotoxicity and carcinogenicity, whereas Cr(III) is far less toxic, due to its poor cellular uptake. However, contradictory results have been published in relation to particulate Cr_2_O_3_. The aim of the present study was to investigate whether Cr(III) particles exerted properties comparable to water soluble Cr(III) or to Cr(VI), including two nano-sized and one micro-sized particles. The morphology and size distribution were determined by TEM, while the oxidation state was analyzed by XPS. Chromium release was quantified via AAS, and colorimetrically differentiated between Cr(VI) and Cr(III). Furthermore, the toxicological fingerprints of the Cr_2_O_3_ particles were established using high-throughput RT-qPCR and then compared to water-soluble Cr(VI) and Cr(III) in A549 and HaCaT cells. Regarding the Cr_2_O_3_ particles, two out of three exerted only minor or no toxicity, and the gene expression profiles were comparable to Cr(III). However, one particle under investigation released considerable amounts of Cr(VI), and also resembled the toxicity profiles of Cr(VI); this was also evident in the altered gene expression related to DNA damage signaling, oxidative stress response, inflammation, and cell death pathways. Even though the highest toxicity was found in the case of the smallest particle, size did not appear to be the decisive parameter, but rather the purity of the Cr(III) particles with respect to Cr(VI) content.

## 1. Introduction

Chromium (Cr) is a naturally occurring element, with three thermodynamically stable forms, namely, Cr(0), Cr(III), and Cr(VI). From a toxicological perspective, the distinction between hexa- and trivalent chromium is of major importance. Exposure to various Cr(VI) compounds has been consistently associated with elevated incidences of respiratory cancers in humans and experimental animals. In contrast, there is no evidence of a carcinogenic action in case of trivalent chromium compounds [1,2,3]. This difference is explained by the so-called uptake-reduction model originally described by Wetterhahn [4]. Cr(VI) ions travel easily through the anion channels of the plasma membrane, and are reduced by intracellular electron donors in three one-electron steps via Cr(V) and Cr(IV), to the stable form of Cr(III). Whereas the anionic chromate is unable to react with DNA directly, Cr(III) forms stable binary (Cr(III)-DNA) and ternary (ligand-Cr(III)-DNA) DNA adducts in Cr(VI) treated cells, where the ligand can be ascorbic acid (Asc), glutathione (GSH), cysteine, or histidine [5]. One proposed outcome of processing the respective DNA lesions is the induction of microsatellite and chromosomal instability [6]. Furthermore, reactive oxygen species are generated in the course of the intracellular reduction of Cr(VI) to Cr(III), leading not only to oxidative stress associated with oxidative DNA damage, but also the activation of redox-regulated signal pathways [7,8]. In addition, epigenetic changes, both on the level of DNA methylation as well as post-translational histone modifications, appear to be associated with Cr(VI) induced carcinogenicity (for a recent review see [9]). It is very likely that a combination of all these mechanisms is involved in Cr(VI)-induced carcinogenicity.

In contrast, the absence of toxic effects in Cr(III) complexes results from their poor ability to enter cells, their lack of intracellular accumulation, and their high stability of coordinated multidentate ligands, which prevent binding to cellular macromolecules (for review see [5]). Nevertheless, contradictory results have been published concerning particulate Cr(III) compounds, which may enter the cell via endocytosis, thereby circumventing the cell membrane barrier reported for water-soluble Cr(III) compounds. Horie and coworkers [10], in particular, demonstrated that in human lung carcinoma A549 cells and human keratinocyte HaCaT cells, Cr_2_O_3_ nanoparticles show severe cytotoxicity, an increase in intracellular reactive oxygen species (ROS) levels, and an activation of antioxidant defense systems and apoptosis; cellular responses were stronger in the Cr_2_O_3_ nanoparticle-exposed cells when compared to cells exposed to micro-sized Cr_2_O_3_ particles or CrCl_3_ [10]. The authors proposed extracellular and/or intracellular Cr(VI) release from nano-sized Cr(III) particles, which needs further clarification, since usually Cr(VI) is reduced in biological media as well as intracellularly to Cr(III). Therefore, the oxidation of Cr(III) particles to Cr(VI) would contradict the current understanding of chromium-induced toxicity, but would be quite important for the toxicological risk assessment of Cr(III) compounds.

Within the present study, we compared two nano-sized and one micro-sized Cr_2_O_3_ particles with respect to cytotoxicity and cellular effects related to genomic stability, and compared it to both K_2_Cr_2_O_7_ and CrCl_3_. Two different cell lines were used, namely the human keratinocyte cell line HaCaT, and A549 human alveolar lung carcinoma cells. The particles were characterized with respect to size, oxidation state, as well as in relation to the release of Cr(III) and Cr(VI) ions in ultrapure water and artificial lysosomal fluid (ALF). Furthermore, besides cytotoxicity, special attention was given to gene expression profiles related to genomic stability, including the genes coding for proteins involved in metal homeostasis, specific DNA repair factors, DNA damage response, oxidative stress response, cell cycle control, and cell proliferation. To this end, a high-throughput RT-qPCR approach was applied as described previously [11]. We observed cytotoxicity and pronounced gene expression alterations, typical for Cr(VI)-induced cellular damage, in the case of water-soluble Cr(VI) and one nano-sized particle. There were very minor, or no effects, in the case of the other two particles under investigation.

## 2. Materials and Methods

### 2.1. Materials

The Cr_2_O_3_ particles were purchased from Nanostructured & Amorphous (Katy, TX, USA) Lot: 1910-091918 (particle A), and Sigma Aldrich (Steinheim, Germany) Lot: 634239 (particle B). Particle C (Lot: CHC 2018-19) was kindly provided by Lanxess (Cologne, Germany). CrCl_3_ hexahydrate (≥97%) and K_2_Cr_2_O_7_ (≥99.5%) were purchased from Carl Roth (Karlsruhe, Germany).

Dimethyl sulfoxide (≥99.9%) and 1,5-diphenylcarbazide (≥97.0%) were purchased from Sigma Aldrich (Steinheim, Germany). A CellTiter-Glo^®^ Luminescent Cell Viability Assay was purchased from Promega (Madison, WI, USA). All PCR consumables, including PCR tubes, strips, reaction tubes, and tubules, as well as cell culture dishes and flasks, were obtained from Sarstedt (Nuembrecht, Germany). The primer pairs were synthesized by Eurofins Genomics (Ebersberg, Germany) or Fluidigm (San Francisco, CA, USA). The DNA suspension buffer, PCR-certified water, and TE buffer were obtained from Teknova (Hollister, CA, USA). The 2X Assay Loading Reagent and 20X DNA Binding Dye Sample Loading Reagent were purchased from Fluidigm (San Francisco, CA, USA). Bio-Rad (Munich, Germany) provided the 2X SsoFastTM EvaGreen^®^ Supermix with Low ROX and the 2X SYBR Green Supermix. The 2X TaqMan^®^ PreAmp Master Mix was obtained from Applied Biosystems (Darmstadt, Germany) and exonuclease I from New England Biolabs (Frankfurt am Main, Germany).

### 2.2. Physicochemical Characterization of Cr_2_O_3_ Particles

#### 2.2.1. TEM

The Cr_2_O_3_ particles were suspended in either sterile ultrapure water or complete media at different concentrations. After sonification, the particle suspensions were applied on copper grids (Plano, Wetzlar, Germany), and then dried prior to analyses. To characterize primary core size, size distribution, and morphological shape, the particles were examined using transmission electron microscopy (CM 200 FEG/ST, Philips, Eindhoven, The Netherlands). ImageJ 1.52d software (U.S. National Institutes of Health, Bethesda, MD, USA) was used to analyze the diameter of individual, non-overlapping particles, and their size distribution was calculated by counting 300 to 500 particles.

#### 2.2.2. Hydrodynamic Size and Polydispersity Index (PDI)

The hydrodynamic size and PDI were determined for particles A and B ( Supplementary Tables S1 and S2). Particle C exerted a very high PDI and sedimented rapidly, which did not allow for respective measurements.

#### 2.2.3. XPS

The XPS measurements were conducted by applying a PHI VersaProbe II system (Physical Electronics PHI/ULVAC-PHI, Chanhassen, MN, USA) equipped with an Al Kα anode (1486.6 eV). For survey spectra, a pass energy of 93.9 eV was used. For all XP detail spectra, the pass energy was set to 23.50 eV. The X-ray power was 100 W and the spot was scanning over an area of 1400 µm × 100 µm. The powder samples were pressed into PTFE cups to achieve a compact and smooth surface. During measurement, a PHI dual beam charge neutralization with low energy Argon ions (~10 eV) and electrons (~2 eV) was used, ensuring a uniform potential without charging effects. Data evaluation was performed using CasaXPS (version 2.3.22, Casa Software Ltd.). All XP spectra in this work were calibrated in relation to the signal of adventitious carbon at 284.8 eV. For the signal fitting, a Shirley background and GL(30) line shapes were used.

#### 2.2.4. Solubility Measurement/Oxidation State

The release of soluble chromium from the Cr_2_O_3_ particles was determined under neutral pH conditions or under acidic pH conditions, the latter resembling conditions in the lysosomes, as previously described [12]. Briefly, stock solutions of 1 mg/mL Cr_2_O_3_ particles were prepared by weighing them into 1.5 mL polystyrene reaction tubes, followed by dilution in 50 mL sterile snap-on lid glasses with either sterile ultrapure water or with artificial lysosomal fluid (ALF), pH 4.5 (composed of sodium chloride (3.210 g/L), sodium hydroxide (6.000 g/L), citric acid (20.800 g/L), calcium chloride dihydrate (0.1285 g/L), disodium hydrogen phosphate (0.0710 g/L), sodium sulphate (0.0390 g/L), magnesium chloride (0.0476 g/L), glycine (0.0590 g/L), sodium citrate dihydrate (0.0770 g/L), sodium tartrate dihydrate (0.0900 g/L), sodium lactate (0.0850 g/L), or sodium pyruvate (0.0860 g/L). The tubes were ultrasonicated for 10 min in a water bath. After 0, 24, 48, or 120 h at room temperature, 1 mL solutions were centrifuged at 16,000× *g* and 4 °C for 1 h. The chromium content was either quantified by the 1,5-diphenylcarbazid (DPC) method or by graphite furnace atom absorption spectrometry (GF-AAS). For the DPC method, 50 µL of reaction mix consisting of 8 µL DPC (1% DPC in acetone), 15 µL sulfuric acid (1 M), 15 µL phosphoric acid (1 M), and 15 µL ultrapure water were added to 200 µL of the sample in a 96-well plate. After shaking for 3 min and incubating for 17 min, the absorption was measured at 540 nm using a multiplate reader TECAN^®^ Infinite M200 Pro (TECAN Group, Maennedorf, Switzerland). Freshly prepared solutions in concentrations of 0, 0.1, 0.2, 0.5, 1.0, and 2.0 mg/L hexavalent chromium (K_2_Cr_2_O_7_ in ultrapure water or ALF, respectively) were used for the calibration.

For the GF-AAS measurement (PinAAcle 900 T, Perkin Elmer, Rodgau, Germany) of the total soluble chromium, 1 mL of the supernatant was heated stepwise to 95 °C to dry up. The remnants were further digested with 1:1 HNO_3_ (69%)/H_2_O_2_ (30%) (*v*/*v*) by repeated stepwise heating to 95 °C. The residue was then solubilized for measurement in 1 mL HNO_3_ (0.2%). The following AAS temperature program was applied: drying at 120 °C for 30 s, and 140 °C for 45 s, 30 s pyrolysis at 1500 °C, atomization at 2300 °C for 5 s, and cleaning for 3 s at 2450 °C.

### 2.3. Cell Culture Experiments

#### 2.3.1. Cr_2_O_3_ Particle Suspensions and CrCl_3_ as Well as K_2_Cr_2_O_7_ Incubation Dilutions

The Cr_2_O_3_ suspensions, as well as soluble Cr(III) and Cr(VI) dilutions, were freshly prepared for each experiment. Particles, received as dry powder, were aliquoted by weighing them into 1.5 mL sterile polystyrene reaction tubes. Watery stock solutions of 1 mg/mL Cr_2_O_3_ were prepared in an endotoxin-free snap-on lid glass by ultrasonication for 10 min. Dilutions in the range of 2, 10, 20, and 50 µg/mL were prepared by adding aliquots of the stock solution into 15 mL sterile falcon tubes filled with adequate volumes of fresh complete medium. Incubation volumes of 200 µL/cm² were chosen to receive particle doses of 0.4, 2.0, 4.0, and 10 µg/cm². Stock solutions of water-soluble CrCl_3_ (200 mM) and K_2_Cr_2_O_7_ (20 mM) were prepared and diluted accordingly, skipping the sonication step. For comparison purposes, the particle suspension of 10 µg/mL (=2 µg/cm²) Cr_2_O_3_ was considered equimolar to 132 µM Cr(III) or Cr(VI).

#### 2.3.2. Cell Culture and Incubation

The human adenocarcinoma cell line A549 (ATCC CCL-185) was kindly provided by Dr. Roel Schins (Leibniz Research Institute for Environmental Medicine, Düsseldorf, Germany). The A549 cells were cultured as a monolayer in RPMI-1640, supplemented with 10% heat-inactivated fetal bovine serum (FBS, Invitrogen, Darmstadt, Germany), 100 U/mL penicillin, and 100 µg/mL streptomycin (both Sigma Aldrich, Steinheim, Germany) at 37 °C in a humidified atmosphere containing 5% CO_2_ (HeraSafe, Thermo Scientific, Langenselbold, Germany). Human keratinocytes HaCaT cells (CLS 300493) were kindly provided by Prof. Dr. Brunhilde Bloemeke (Trier University, Department of Environmental Toxicology, Trier, Germany). The cells were cultured at 37 °C, 5% CO_2_ in a humidified atmosphere in Dulbecco’s modified Eagle’s medium (DMEM, Sigma Aldrich, Steinheim Germany), supplemented with 10% FBS (Invitrogen, Darmstadt, Germany), 100 U/mL penicillin, 100 µg/mL streptomycin (both Sigma Aldrich, Steinheim, Germany), and 2 mM GlutaMAX™ (Gibco, Carlsbad, CA, USA). Both cell lines were grown up to 80% confluency and routinely split three times per week. Passage numbers from 8 to 25 (HaCaT) and from 14 to 35 (A549) were used for experiments. The measurement of the ATP content was carried out in white-walled optical-bottom 96-well plates (ThermoFisher, Dreieich, Germany). Seeding density was either 1 × 10^4^ cells/well for HaCaT, or 3 × 10^4^ cells/well for A549 cells. For gene expression analyses 0.5 × 10^6^ were seeded in 6 cm cell culture dishes (Sarstedt, Nuembrecht, Germany). After 24 h (A549) or 48 h (HaCaT) the supernatant was removed from the logarithmically growing cells and was replaced by the particle suspension or Cr(III)/(VI) dilution. For consistent particle deposition, the incubation volume for each experiment was set at 0.2 mL per square centimeter growth area.

#### 2.3.3. Cytotoxicity Assay

A Promega CellTiter-Glo^®^ ATP assay was used to analyze cell viability and cell proliferation. Logarithmically growing cells were incubated for 24 h with 0.4, 2.0, 4.0, or 10.0 µg/cm² Cr_2_O_3_ particles; 26.4, 66.0, 132, 264, 660, or 1320 µM (not shown) CrCl_3_; or 1.32, 2.64, 6.6, 13.2, 26.4, or 66 µM K_2_Cr_2_O_7_. The incubation solution was removed after 24 h and the cells were washed twice with PBS. The CellTiter-Glo^®^ Luminescent Cell Viability Assay was carried out according to the manufacturer’s protocol. Briefly, complete medium and the equal volume of CellTiter-Glo^®^ reagent were added to the cavities. The 96-well plate was transferred on an orbital shaker for 2 min to induce cell lysis. The plate was incubated for 40 min in the dark at room temperature to stabilize the luminescent signal, which was then recorded with a microplate reader TECAN^®^ Infinite M200 Pro (TECAN Group, Maennedorf, Switzerland). Data were analyzed with Excel 2010 (Microsoft, Redmond, CA, USA). The ATP content as a measure for cell viability was expressed as percentage normalized to non-treated control cells. To test if soluble Cr(III) or Cr(VI) interfered with the ATP assay, the relevant concentrations from above were added to a standard curve of ATP (0.5, 1.0, 2.5, 5.0 µM).

#### 2.3.4. Gene Expression Analyses

Gene expression analyses via high-throughput RT-qPCR using the Fluidigm dynamic array on the BioMark™ System were performed as described by Fischer et al. [11]. Briefly, 0.5 × 10^6^ cells were treated with different concentrations of Cr_2_O_3_ particles, CrCl_3,_ or K_2_Cr_2_O_7_ in complete medium. After 24 h, the cells were washed in PBS, trypsinized, and resuspended in ice-cold PBS containing 10% FBS, and collected by centrifugation. RNA isolation was performed using MN NucleoSpin^®^ RNA Plus KIT (Macherey-Nagel, Dueren, Germany), according to the manufacturer’s protocol. Then, 1 µg of RNA was reverse transcribed in duplicates into complementary DNA (cDNA) using qScript™ cDNA Synthesis Kit (Bio-Rad, Munich, Germany). Subsequently, a specific target amplification (STA) and an exonuclease I digestion (EXO) were performed prior to qPCR. Then, 5 µL of the STA and EXO mix containing 1.25 µL cDNA mix from cDNA synthesis, 0.5 µL pooled primer mix (PPM), 2.5 µL 2X TaqMan^®^ PreAmp Master Mix, and 0.75 µL PCR-certified water were applied on the Fluidigm dynamic array. Controls, such as a no-template control (NTC-STA) and a non-reverse transcribed RNA control (NoRT) were considered. All pipetting steps, until reverse transcription, were performed under a sterile RNA hood. Pipetting the cDNA was carried out under a DNA/DNase-free hood, to prevent cross-contamination during the entire qPCR experiment. Any details regarding various temperature profiles for RT, STA, EXO, qPCR, and melting curve analyses can be found in the original publication from Fischer et al. [11]. Preparation and loading of Fluidigm 96.96 Dynamic Array IFC (integrated fluidic circuit) were performed according to the manufacturer’s instructions. After priming, the chip was loaded with samples and primer reaction mixes within 1 h to prevent unfavorable evaporation effects and loss of pressure. Samples and primer reaction mixes were loaded into the chip by running the Load Mix (136×) script of the IFC Controller HX (Fluidigm, San Francisco, CA, USA). The chip was transferred into the BioMark™ System (Fluidigm, San Francisco, CA, USA) immediately, and qPCR and subsequent melting curve analyses were performed. Data analysis was executed with the Fluidigm Real-Time PCR Analysis tool and GenEx^TM^ 5 software (MultiD Analyses AB, Gothenburg, Sweden). Transcription levels of five reference genes (*ACTB*, *B2M*, *GAPDH*, *GUSB*, and *HPRT1*) were used for normalization. Alterations in transcript levels of the target genes were displayed as a log_2_ fold change compared to a control group by calculating relative quantities corresponding to the ΔΔCq method [13,14].

#### 2.3.5. Statistics

If not stated otherwise, all data are displayed as the mean of three independently performed experiments, each of which was conducted at least in duplicates. For cell viability, differences on the cellular level between the negative control (non-treated cells) and the metal compound treatment were analyzed using one-way ANOVA followed by a Dunnett’s T post hoc test.

## 3. Results

### 3.1. Particle Characteristics

Three different Cr_2_O_3_ particles were included, two in the nano-sized range and one in the micro-sized range. Particle A was nano-sized and was obtained from the same supplier as stated in the study of Horie and coworkers [10].

**Figure 1:**
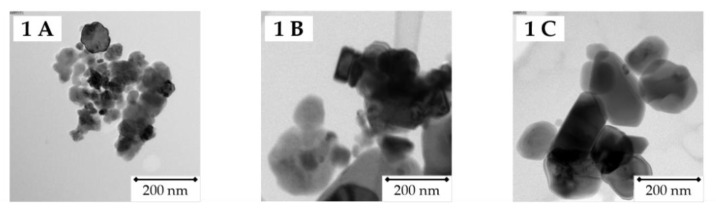

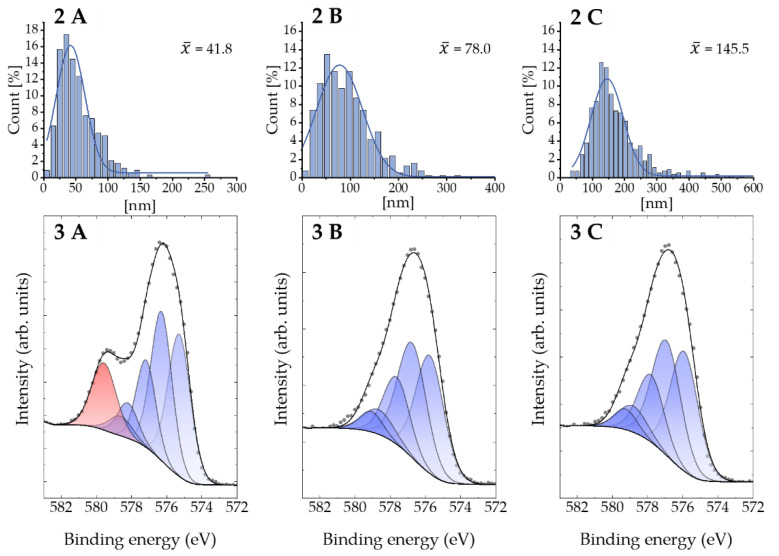
Physicochemical characterization of different Cr_2_O_3_ particles. Size distribution was measured in ultrapure water at a concentration of 10 µg/cm². **1A**–**1C**: representative transmission electron microscopy (TEM) images of the particles. **2A**–**2C**: average diameters of particle A (42 nm), B (78 nm) and C (146 nm). **3A**–**3C**: X-ray photoelectron spectroscopy (XPS) spectra of the particles. The red signal at 579.6 eV only present in Figure 1(**3A**) is characteristic for Cr(VI), while the blue multiplet signal between 575.3 and 578.6 eV is characteristic for Cr(III).

No differences in morphology were detected between the particles. Particle size was determined using transmission electron microscopy (TEM). Representative TEM images of the particles are shown in Figure 1(1). Particles A and B are nano-sized, with average diameters of 40 nm and 80 nm, respectively. Particle C is micro-sized, with an average diameter of 150 nm (Figure 1(2)).

The oxidation state of the Cr_2_O_3_ particles was measured using X-ray photoelectron spectroscopy (XPS), and detailed spectra of chromium 2p_3/2_ are shown for the three different particles (Figure 1(3)). Particles B and C show relatively pure Cr(III) multiplets spectra (shown in blue), as expected for Cr_2_O_3_ [15]. Compared to values in the literature [15], the peaks are broadened, which is most likely due to the powder nature of the sample. However, in contrast to particles B and C, particle A clearly shows a contribution of Cr(VI) to the spectrum (red signal), accounting for about 15% of the total chromium content.

### 3.2. Cytotoxicity

In the next step, the cytotoxicity of all three particles was investigated, as well as water-soluble Cr(III) and Cr(VI), by applying the CellTiter-Glo^®^ luminescent cell viability assay (Promega). This assay is based on the quantification of ATP after complete cell lysis, via the reaction of beetle luciferin to oxyluciferin and photons. The luminescence generated correlates with the ATP content, and therefore, the number of viable cells in culture.

**Figure 2:**
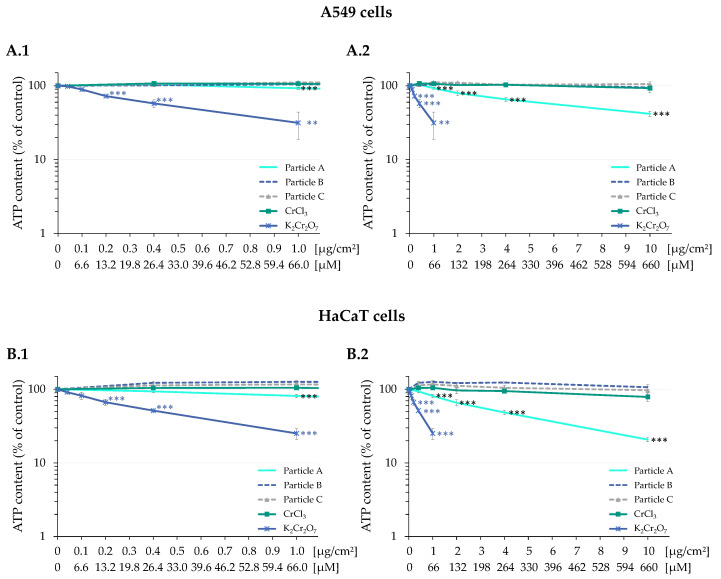
ATP content of A549 (**A**) and HaCaT (**B**) cells after 24 h treatment with Cr(III) oxide particles Cr_2_O_3_, CrCl_3_, or K_2_Cr_2_O_7_. Treatments below 66 µM (corresponding to 1 μg/cm^2^ chromium) are shown in (**A.1**) and (**B.1**). Treatments covering the entire dose range investigated are depicted in (**A.2**) and (**B.2**). Mean values ± standard deviations derived from three independent experiments are shown. Statistics were performed using ANOVA followed by Dunnett’s T post hoc test: ** *p* ≤ 0.01, *** *p* ≤ 0.001.

The cytotoxicity of K_2_Cr_2_O_7_, CrCl_3_, and the three different Cr_2_O_3_ particles was measured after 24 h incubation. Results for both cell lines are shown in Figure 2A,B. Treatment with K_2_Cr_2_O_7_ exerted pronounced dose-dependent cytotoxicity starting in the low micromolar concentration range; the ATP content was reduced by 11% (A549) or 18% (HaCaT) after treatment with 6.6 µM, and by 42% (A549) or 49% (HaCaT) after treatment with 26.4 µM. Severe cytotoxicity was observed after treatment with Cr(VI) concentrations of 66 µM and above. In case of CrCl_3_, neither differences in confluency nor a reduction in ATP content were evident at concentrations up to 264 µM. Treatment with 660 µM showed a minor reduction of the ATP content by 20% in HaCaT cells and 10% in A549 cells. Concerning the three different Cr_2_O_3_ particles, only particle A showed cytotoxic effects at doses above 1 µg/cm². As displayed in Figure 2A, the viability of the A549 cells decreased by 35% after incubation with 4 µg/cm², and by 58% after incubation with 10 µg/cm². ATP depletion was even more pronounced in HaCaT cells (Figure 2B). Here, viability was reduced to 81%, 65% and 21% at 2, 4 and 10 µg/cm² Cr_2_O_3_, respectively. In contrast, no significant cytotoxicity was observed in either cell line for particles B or C.

### 3.3. Release of Soluble Chromium from Cr_2_O_3_ Particles

**Figure 3:**
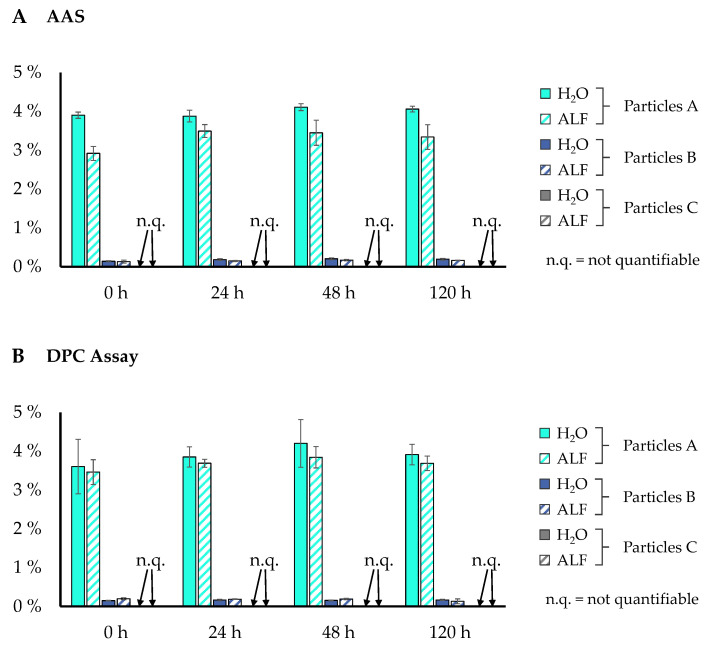
Release of chromium from Cr_2_O_3_ particles in different media. Cr_2_O_3_ particles measuring 1.0 mg/mL were either incubated in ultrapure water (pH 7.0) or artificial lysosomal fluid (ALF) (pH 4.5) for 0 h, 24 h, 48 h, or 120 h. The remaining particles were removed from the supernatant by centrifugation as described in Materials and Methods. Total chromium release was quantified by atomic absorption chromatography (AAS) (**A**) or Cr(VI) release by a colorimetric DPC assay (**B**) by applying the chromogenic dye 1,5-diphenylcarbazone. Mean values of 3 independent determinations ± SD are shown.

Since particle A showed considerable cytotoxicity, while neither CrCl_3,_ nor particles B and C were cytotoxic, we investigated whether this may be due to Cr(VI) release as indicated by our XPS studies. As a first step, chromium release from the particles was determined via AAS to quantify the content of all soluble chromium species in supernatants. For this purpose, 1 mg/mL particles were either suspended in ultrapure water (pH 7.0) or artificial lysosomal fluid (ALF) with a pH of 4.5 (both at room temperature), to simulate potential intracellular chromium release after endocytosis within the lysosomes. Supernatants were centrifuged at 16,000× *g* for 1 h before measurement. To investigate a potential time dependency of chromium release, the measurements were performed immediately after the preparation of the suspension (0 h), after 24 h, 48 h, or 120 h as depicted in Figure 3A. The results obtained by AAS demonstrated an immediate chromium release from particle A of around 4% in ultrapure water, with no considerable increase in time. This fraction was a little less pronounced in ALF, reaching around 3.5%. Chromium release from particle B was significantly lower, resulting in 0.18% in ultrapure water and 0.15% in ALF. Chromium release from particle C was not quantifiable in a reproducible manner.

To differentiate between the release of Cr(VI) and Cr(III), a colorimetric assay was applied. Due to the tetrahedral structure of Cr(VI), it is prone to form complexes with chromogenic dyes, such as 1,5-diphenylcarbazone (DPC), which forms red–violet products, proportional to the amount of Cr(VI) present in the sample. As shown in Figure 3B, a Cr(VI) content of 3.9% for particle A, and 0.16% in case of particle B, was detected in ultrapure water after 24 h. For particle C, the chromium content was below the limit of quantification of 0.038 mg/L. As previously, particle suspensions were measured after 0 h, 24 h, 48 h, and 120 h to examine a time-dependent dissolution of the particles, and a potential release of Cr(VI) in ultrapure water and ALF. As depicted in Figure 3A,B, chromium was detectable immediately after contact with either ultrapure water or ALF, while no significant pH or time dependency was observed.

### 3.4. Gene Expression Analysis

**Figure 4:**
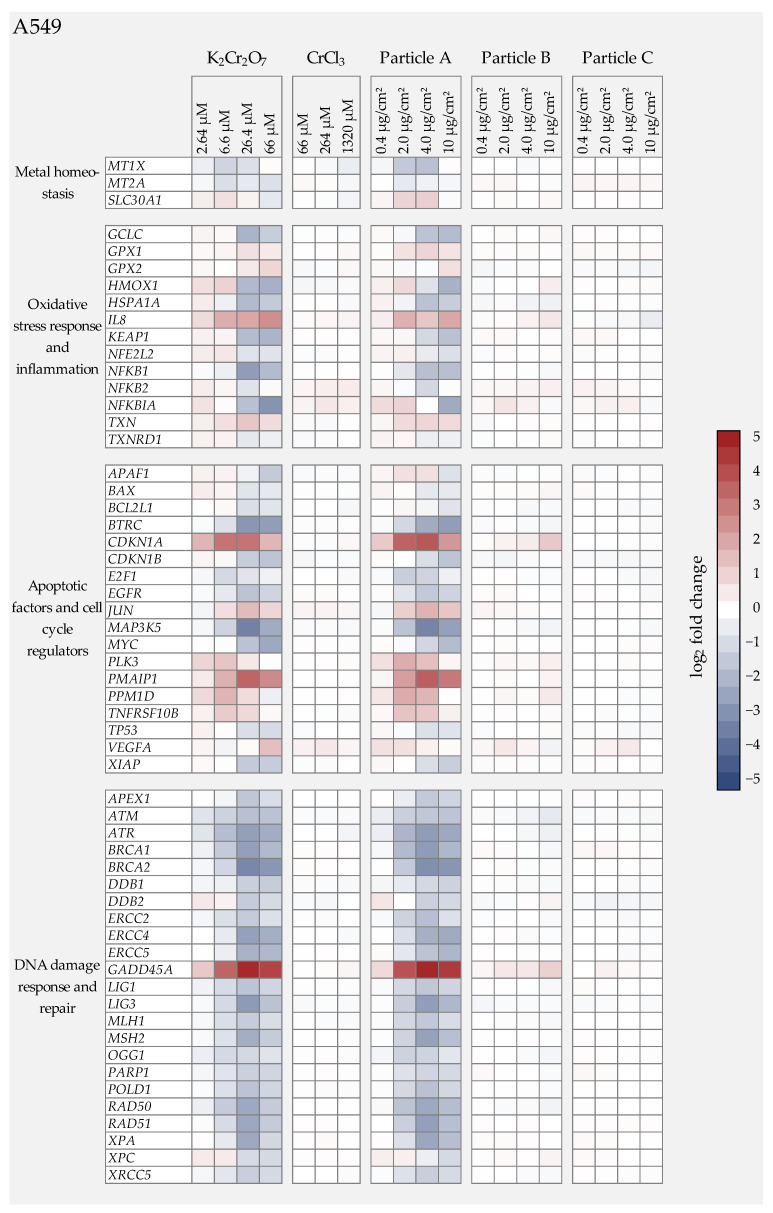
Overview of the impact of K_2_Cr_2_O_7_, CrCl_3_, or three different Cr(III) oxide particles on human lung epithelial cells (A549) using a high-throughput RT-qPCR approach with a custom-designed gene set. The genes under investigation have been clustered into groups associated with metal homeostasis, oxidative stress response, inflammation, apoptosis, and cell cycle regulation as well as DNA damage response and repair. A549 cells were treated with the respective chromium compound for 24 h. Displayed are the log_2_ fold changes of relative gene expression as a heatmap. Red colors indicate an enhanced expression, and blue colors indicate a down-regulation. The mean values of at least three independently conducted experiments are shown.

**Figure 5:**
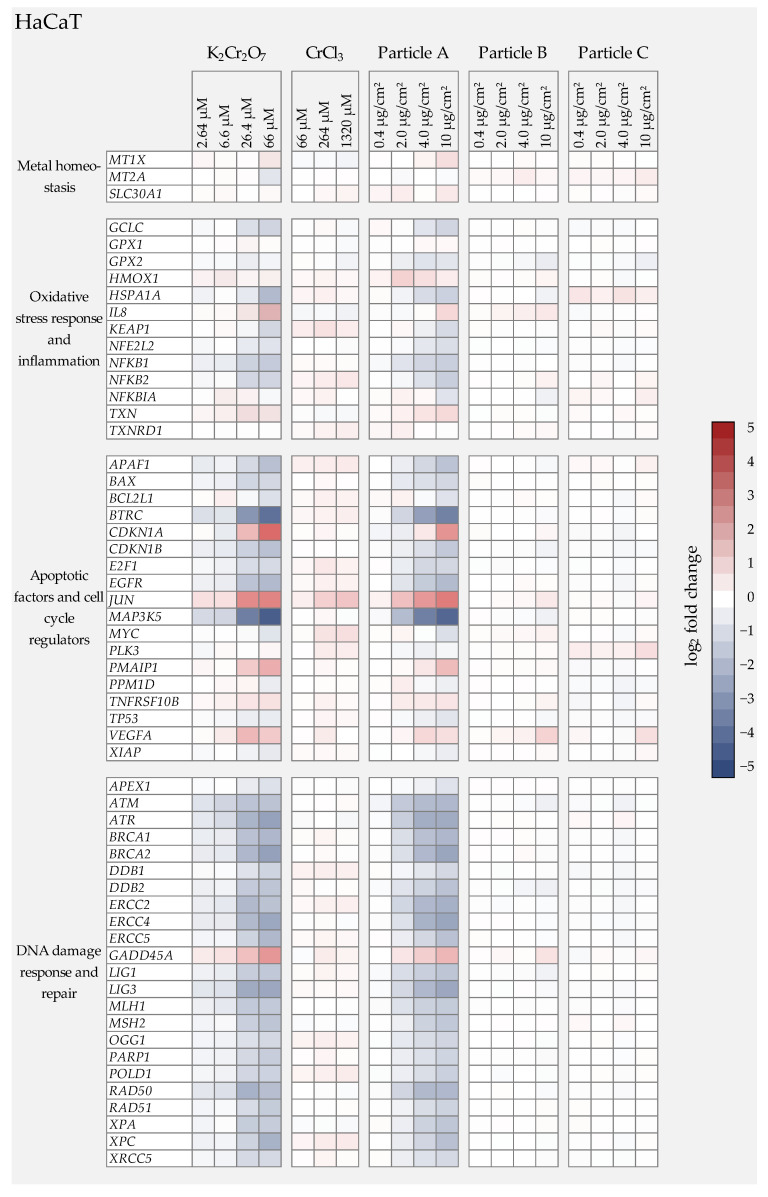
Overview of the impact of K_2_Cr_2_O_7_, CrCl_3_, or three different Cr(III) oxide particles on human keratinocytes (HaCaT) using a high-throughput RT-qPCR approach with a custom-designed gene set. The genes under investigation have been clustered into groups associated with metal homeostasis, oxidative stress response, inflammation, apoptosis, and cell cycle regulation as well as DNA damage response and repair. HaCaT cells were treated with the respective chromium compound for 24 h. Displayed are the log_2_ fold changes of relative gene expression as a heatmap. Red colors indicate an enhanced expression, and blue colors indicate a down-regulation. The mean values of three independently conducted experiments are shown.

As described in the introduction, Cr(VI) has been shown consistently to induce DNA damage as well as oxidative stress, while Cr(III) is considered to be largely non-toxic due to a very limited uptake in cells. Nevertheless, whether or not this applies also to Cr(III) oxide particles, which can enter the cells via endocytosis, remains to be elucidated. In the present study, therefore, we aimed to obtain toxicity profiles for all three particles and to compare them with water-soluble Cr(VI) and Cr(III). To this end, we applied gene expression analyses using high-throughput RT-qPCR, established previously in our group using the BioMark HD system [11], and which has been used successfully for metal-based nanomaterials, both after submersed and air-liquid interface (ALI) exposure [16,17,18]. This method enables the parallel investigation of 96 samples with regard to their impact on 95 genes of interest. Within our system, the genes were selected to yield expression profiles related to genomic stability, and can be grouped into six gene clusters: xenobiotic metabolism, metal homeostasis, (oxidative) stress response and inflammation, DNA damage response and repair, cell cycle regulation, and apoptosis. A complete list of genes and their encoding proteins is provided in Supplementary Table S3. For better visualization, changes in transcription were calculated as fold_2_ changes of expression levels. A reduction of at least 50% (log_2_ fold change ≤ −1) or a doubling (log_2_ fold change ≥ 1) were considered relevant when compared to the respective control [16]. Within these investigations, the impact of K_2_Cr_2_O_7_, CrCl_3_, and the three Cr_2_O_3_ particles on gene expression profiles was examined in both cell lines after 24 h incubation. Based on the cytotoxicity data shown in Figure 2, particle doses of 0.4, 2.0, 4.0, and 10.0 µg/cm² were chosen and compared to toxicity profiles at 2.64, 6.6, 26.4, and 66 µM Cr(VI) and 66, 264, and 1320 µM CrCl_3_. The heatmaps in Figure 4 and Figure 5 summarize expression patterns of those genes for which expression alterations were considered relevant. A complete overview of the results obtained for the entire gene set is provided in Supplementary Figures S1 and S2. For both cell lines, a strong impact of Cr(VI) was observed in the gene clusters of DNA damage response, cell cycle regulation, and apoptosis.

#### 3.4.1. Water Soluble Cr(VI) Treatment

Within the applied dose range of 2.64 to 66 µM, treatment with K_2_Cr_2_O_7_ elicited some distinct changes in gene expression. As a general picture, both cell lines showed similar gene expression profiles, with some differences for individual genes and dose-dependencies (Figure 4 and Figure 5).

In A549 cells, the strongest impact on gene expression was seen after treatment with 26.4 µM Cr(VI). Genes related to DNA damage response and repair were affected the most. Thus, the DNA damage response gene *GADD45A* (growth arrest and DNA damage-inducible gene α) showed a pronounced dose-dependent increase starting at the lowest, non-cytotoxic concentration, and reached induction levels up to a 28-fold expression change. This was also the case for the HaCaT cells, although to a slightly lesser extent. Interestingly, the expression of specific DNA repair proteins was downregulated in both cell lines, particularly in case of *ATR*, *BRCA1*, *BRCA2*, *ERCC4*/*XPF*, *MLH1, MSH2, LIG3*, *RAD50*, *RAD51*, and *XPA,* which are involved in all major DNA repair pathways. Once again, effects started at the lowest dose and transcription levels were reduced by up to 80% at cytotoxic concentrations. Even though the gene expression pattern was similar in both cell lines, respective alterations occurred at lower concentrations in the A549 cells.

In the cluster of oxidative stress response and inflammation, in both cell lines a concentration-dependent and relevant induction was only evident for *IL8* (Interleukin 8), which is involved in the inflammatory response. Several other genes such as heme oxygenase 1 (*HMOX1*) were slightly up-regulated at low concentrations, but down-regulated at higher concentrations. Again, effects were more pronounced in the A549 cells when compared to the HaCaT cells. K_2_Cr_2_O_7_ further down-regulated the expression of antioxidant responsive genes such as *GCLC* (γ-Glutamyl cysteine synthetase), *NFkB1,* and *NFkB1A* in the A549 cells, as well as *NFkB1* and *NFkB2* in the HaCaT cells.

With regard to cell cycle regulators, one of the most striking effects was the dose-dependent up-regulation of *CDKN1A* in both cell lines, which encodes the protein p21. In addition to its involvement in cell cycle arrest upon DNA damage, it plays an important role in DNA repair, DNA replication, and apoptosis.

Regarding genes related to specific cell death pathways, in A549 and to a lesser extent HaCaT cells, the induction of *PMAIP*, which encodes the proapoptotic BCL-2 protein Noxa, was observed, predominantly at higher concentrations. In contrast, the strongest gene repression was exhibited by the gene *MAP3K5* coding for Ask1 (apoptosis signal-regulating kinase 1); low dose treatment with 6.6 µM decreased the transcription rate of this gene by more than 50% in A549 and HaCaT cells. Similarly, *BTRC* (beta-transducin repeat containing E3 ubiquitin-protein ligase) was strongly repressed in A549 and HaCaT; it also plays a key role in apoptosis. Finally, *JUN* coding for the transcription factor c-Jun was up-regulated in both cell lines; after incubation with 26.4 µM, the transcription increased three-fold (A549) and five-fold (HaCaT).

#### 3.4.2. Water Soluble Cr(III) Treatment

The gene expression patterns of CrCl_3_ differed strongly from those of K_2_Cr_2_O_7_. In general, the applied Cr(III) treatments exhibited no significant modulations of the genes under investigation over the complete concentration range.

#### 3.4.3. Cr(III) Oxide Particle Treatment

In direct comparison with the gene expression results of the soluble Cr(VI) compound, it was noticeable that the gene expression patterns of the K_2_Cr_2_O_7_ treatment and those of the Cr(III) oxide particle A treated cells were almost identical, suggesting that the cellular effects were solely due to the release of Cr(VI).

Particle B exhibited almost no changes in gene expression. Only in the A549 cells were *GADD45A* transcript levels very slightly elevated, signaling low levels of DNA damage, as well as *CDKN1A* involved in cell cycle arrest, both apparent at the highest dose level. No relevant effects were observed in the HaCaT cells.

Finally, particle C showed no relevant alterations in gene expression profiles in either cell line within the applied dose range of 0.4 µg/cm² to 10 µg/cm².

## 4. Discussion

Based on the publication of Horie et al. [10] we aimed to investigate whether Cr(III) particles—especially those in the nano-sized range—exerted properties of Cr(VI), due to either intracellular Cr(III) release with the subsequent induction of DNA damage, or via the release of Cr(VI) either extracellularly or intracellularly. This question is of utmost importance for toxicological risk assessment, since Cr(VI) is considered to be carcinogenic, and diverse mechanisms, including the induction of DNA damage, have been identified to contribute to carcinogenicity. On the other hand, Cr(III) is considered to be far less toxic and/or genotoxic, but clarification is still needed whether this also applies to nano-sized Cr(III) particles.

In addition to the determination of cytotoxicity, we used a very sensitive high-throughput RT-PCR method [11,16,17] as a central approach, to establish the toxicological fingerprints of water-soluble Cr(VI) and Cr(III), as well as those of three different Cr_2_O_3_ particles differing in size and manufacturer. While gene expression profiles of Cr(VI) clearly identified the induction of DNA damage, and to some extent also the induction of oxidative stress, water soluble Cr(III) provoked no changes in gene expression profiles up to millimolar concentrations, in agreement with its low toxicity. Regarding the Cr_2_O_3_ particles under investigation, our results confirm in principle the cellular damage provoked by particle A, which was apparent also in the Horie study [10]. The cause for the observed damage appears to be the Cr(VI) present in the particles, as well as its subsequent release. Only very minor effects were observed in the case of the nano-sized particle B, whereas no corresponding effects were apparent for particle C, which was micro-sized.

Within the present study, K_2_Cr_2_O_7_ exerted the highest toxicity, causing severe depletion of ATP content in both cell lines in the low micromolar concentration range. CrCl_3,_ on the other hand, had no significant impact on the metabolic activity and did not decrease the ATP levels in either the A549 or HaCaT cells. A study by Hininger and coworkers revealed similar results for the cytotoxicity of water-soluble Cr(III) and Cr(VI) in HaCaT cells, determined by MTT conversion and LDH release. Even though the toxicity endpoints differ and a direct comparison of the cytotoxic effect requires some adjustment, Cr(VI) was found to be strongly cytotoxic at low micromolar concentrations, whereas Cr(III) chloride exerted toxicity only in the millimolar range [19].

These differences are due to the considerably higher permeability of cell membranes in relation to Cr(VI) when compared to Cr(III). As described extensively in the literature, Cr(VI), due to its tetrahedral configuration, enters the cell via sulfate or phosphate ion channels, whereas the permeability of cell membranes in relation to Cr(III) is much lower [4,20]. The toxicity of Cr(VI) is attributed to its intracellular reduction to Cr(III). In the course of reduction, highly reactive chromium intermediates Cr(V) and Cr(IV) are generated, concurrent with the formation of reactive oxygen species (ROS), that mediate the oxidation of molecular targets, including membrane-associated phospholipids, proteins, and DNA [21,22]. The final intracellular reduction product is Cr(III), which forms stable binary (Cr(III)-DNA) and ternary (ligand-Cr(III)-DNA) adducts, the latter with ascorbic acid (Asc), glutathione (GSH), cysteine, or histidine as ligand, depending on the availability of intracellular reductants [5].

With regard to the three different Cr_2_O_3_ particles under investigation, only particle A exerted toxicity in the applied dose range, whereas there was no reduction in ATP content detectable for either the nano-sized particle B, nor for the micro-sized particle C. Therefore, even though particle A was the smallest, the size of the particles is not the effective parameter explaining the toxicity. When comparing both cell lines, the HaCaT cells showed a more pronounced reduction in ATP content, and therefore, in cell viability after exposure towards particle A and soluble Cr(VI). This is consistent with results published by Horie and coworkers. In their study, the same particles were investigated, also using HaCaT and A549 cells, even though different toxicological endpoints (MTT conversion and LDH release), higher Cr_2_O_3_ concentrations, and shorter incubation times were applied [10].

These differences in cytotoxicity of the three particles under investigation were further elucidated. Since neither particle B, which is nano-sized like particle A, nor particle C in the micro-sized range was cytotoxic, the toxicity was not related to the size of the particles. Therefore, differences in particle toxicity were most likely related to the Cr(VI) content. As a first step, the oxidation state of the Cr_2_O_3_ particles was measured using X-ray photoelectron spectroscopy (XPS.) While particles B and C exerted relatively pure Cr(III) multiplets spectra as expected for Cr_2_O_3_, particle A clearly revealed a considerable Cr(VI) content of about 15%, based on the total chromium content. This raised the question whether Cr(VI) is released from the particles, and if so, whether or not Cr(VI) is expected to be released extracellularly at a neutral pH, or whether it is more likely to be released intracellularly, for example after endocytic uptake of the particles into lysosomes, under acidic pH conditions. To discriminate between these possibilities, the release of total chromium under both conditions was investigated via AAS, as a first step. In the second step, a colorimetric Cr(VI)-specific assay was applied to distinguish between the two chromium species. The Cr_2_O_3_ particles were dispersed for up to 120 h in either ultrapure water or in ALF (pH 4.5). After centrifugation at 16,000× *g*, the supernatants were analyzed accordingly. Soluble chromium released from particle C was neither quantifiable by AAS, nor within the Cr(VI) specific DPC assay. Particle B showed a moderate chromium release of 0.18%, which was also detected in its entity by the colorimetric approach (0.19%), and thus, identified as Cr(VI). Particle A, however, showed a pronounced chromium release of around 4%, detected by AAS immediately after dissolution in water, with no time-dependent increase. Furthermore, experiments with the chromogenic dye DPC revealed that the chromium released from particle A was almost exclusively Cr(VI), amounting to 3.6%. Compared to the total chromium content of 15%, as determined by XPS (Figure 1(3)), it is evident that Cr(VI) was not released completely. This fraction was also not increased in artificial lysosomal fluid when compared to water. These data provide evidence that particle A immediately released considerable amounts of Cr(VI) extracellularly under neutral pH conditions.

Our results indicate that the release of chromium strongly depends on the specific particles under investigation. This confirms published results, even though only few studies discriminated between Cr(VI) and Cr(III). Even studies conducted with particles from the same manufacturer have shown large deviations when assessing chromium release, also depending on the respective method of quantification. Horie et al., for example, observed 1.0–1.5% soluble chromium with 0.4% Cr(VI) in supernatants of particle A suspensions after ultrafiltration and centrifugation in complete media containing FBS [10]. Another group using nano-sized Cr_2_O_3_ from the same manufacturer detected a total chromium release of up to 0.15% with 0.09% Cr(VI) from particles suspended in artificial wastewater (100 mg/L) [23]. Nanoparticles from other suppliers also differed with respect to chromium release. While Kumar et al. [24] could not detect any release of chromium via AAS under their test conditions, Peng and colleagues found 4.1% soluble chromium after 24 h and 5.4% after 48 h in a saline buffered solution [25]. Both studies used Cr_2_O_3_ NP from different manufacturers, and did not distinguish between the chromium species. In another study conducted by Costa et al. particle suspensions of 1.0 and 10.0 g/L were analyzed, reporting a total chromium release of approximately 0.15%, with a Cr(VI) content of up to 0.08%. [26].

These findings raise the question why in some cases Cr(VI) is released from Cr_2_O_3_ particles, while in other cases no chromium release is observed at all. This phenomenon could be caused by differences in production processes of the respective Cr_2_O_3_ particles. They are produced by the reduction of alkali chromates. To obtain pure Cr_2_O_3_, a complete reduction of chromates, with a subsequent purification process, is mandatory. Production processes with incomplete reductions of alkali chromates, or insufficient purification steps, result in detectable amounts of Cr(VI). This may be more pronounced in the case of nano-sized particles [27]. Therefore, under toxicological considerations, a characterization of the particles including Cr(VI) release prior to use is of utmost importance.

Within this study, to the best of our knowledge, a quantitative high-throughput RT-qPCR method was applied for the first time to elucidate the toxicological impact of the three Cr_2_O_3_ nano- and micro-sized particles in comparison with water-soluble Cr(VI) and Cr(III). Since Cr(VI) is carcinogenic, via the induction of DNA damage as the primary mode of action, special emphasis was given to genes involved in DNA damage signaling, DNA repair, cell cycle control, and apoptosis. Furthermore, potential effects on the oxidative stress response were considered. In principle, cellular alterations of gene expression patterns may result from Cr(VI) released by the particles, but could also be the consequence of uptake via endocytosis, followed by intracellular chromium release and the formation of DNA adducts typical for Cr(III). The investigations of water-soluble chromium compounds clearly shows significant differences in the effect of Cr(III) and Cr(VI). Even low micromolar concentrations of Cr(VI) resulted in altered gene expression in the clusters of DNA damage response, cell cycle regulation, and cell death pathways, as well as in—even though less pronounced—oxidative stress response. Cr(III), on the other hand, showed no relevant changes in gene expression patterns, even after treatment with millimolar concentrations.

As a predominant mode of action, Cr(VI) induces DNA damage after intracellular reduction. Besides the formation of highly reactive Cr(V) and Cr(IV) intermediates, which may lead to the formation of ROS, stable binary (Cr(III)-DNA) and especially ternary Cr(III)-DNA adducts are generated, involving cellular reducing agents such as ascorbate or glutathione [5,9,28]. One proposed outcome of processing the respective DNA lesions is the induction of microsatellite and chromosomal instability [5]. With regard to the gene expression profiles obtained for Cr(VI) within the present study, the most eminent outcome was the induction of the DNA damage signaling gene *GADD45A* in both cell lines. Furthermore, the cell cycle regulator *CDKN1A* was induced, coding for p21, and stabilizing p53 upon the induction of DNA damage, with a subsequent cell cycle arrest [29]. In contrast, the expression of several specific DNA repair factors was down-regulated. The inhibited transcription was particularly pronounced in case of *ATR*, *BRCA1, BRCA2*, *ERCC4/XPF*, *MLH1*, *MSH2*, *LIG3*, *RAD50*, *RAD51*, and *XPA*, coding for enzymes and proteins involved in DNA double-strand break (DSB) repair (*ATR*, *BRCA1*, and *BRCA2*, *LIG3*, *RAD50*, *RAD51*), nucleotide excision repair (*XPA* and *ERCC4*/*XPF*) or mismatch repair (*MLH1* and *MSH2*).

The down-regulation of DNA repair genes appears to be an important mechanism in order to modulate cellular DNA repair capacity. In this context, epigenetic changes play an important role in gene regulation, and likely contribute to carcinogenicity [30]. In some studies, the interaction of Cr(VI) with proteins related to transcription, or its interference with epigenetic modulators, was also demonstrated and associated with genomic instability [9,30,31]. Thus, many of the not yet fully understood toxic effects of Cr(VI), such as Cr(VI)-induced cellular DNA repair deficiencies or Cr(VI)-induced genomic instability, are currently thought to be due to epigenetic changes [6,32]. Impaired transcription can occur due to different reasons. Thus, promotor regions of genes can accumulate 5-methyl cytosine (5-mC) in guanine- and cytosine-rich regions (CpG islands), which are predominantly found in close distance to the transcription starting site. For instance, in Cr(VI)-exposed lung epithelial cells, a sequence-specific increase of 5-mC led to the down-regulation of DNA repair genes [31,33]. Furthermore, hypermethylation of the *MLH1* gene has been linked to a defective mismatch repair, resulting in microsatellite instability in lung tissue of Cr(VI)-exposed workers [34,35]. Also, recently published transcriptome studies support the hypothesis that Cr(VI) may have a pronounced effect on the transcriptional response via a variety of epigenetic modifications, contributing to carcinogenesis [32]. In addition to altered DNA methylation patterns, there is some evidence that chromates lead to a decreased transcription rate via changes in histone modifications, by interference with enzymes involved in the acetylation and methylation of histone side chains. Thus, in a study conducted by Sun et al. exposure of A549 and BEAS2B cells to Cr(VI) resulted in a decrease in histone modification H3K27Me3, and an increase in modifications H3K4Me3, H3K9Me2, and H3K9Me3 [36]. In particular, methylation at H3K9 or H3K27 is closely associated with transcriptional repression [37].

One other postulated mechanism of Cr(VI) induced cellular toxicity is the generation of ROS. Thus, after the anion carrier-mediated uptake of Cr(VI) [38,39], it is reduced intracellularly to the highly reactive chromium intermediates Cr(V) and Cr(IV), causing oxidative stress [5,9,37]. However, our data do not seem to support the extensive generation of ROS. At low concentrations, only marginal effects were observed in the gene cluster of oxidative stress response. One exception was *IL8*, signaling inflammation, which was induced in both cell lines. At higher concentrations, Cr(VI) ions instead caused a repression of oxidative stress-related genes, such as *NFKB1* and *NFKB2*. These observations appear to contradict results reported by Ye and Shi [40], who described elevated transcription levels of glutathione peroxidase (*GPx*), copper-zinc superoxide dismutase (*SOD*), and metallothionein 2A (*MT2A*), as well as the metal-regulatory transcription factor 1 (*MTF1*) in A549 cells. However, in their study, two hour, high-dose (K_2_Cr_2_O_7_, 300 µM) treatments were applied. With respect to particle A, the data of Horie and colleagues also indicated an increased formation of ROS. However, in this case, ROS were not detected at the transcriptional level, but were measured directly by the colorimetric DCFH assay. By this approach, a clear increase of the intracellular ROS was observed in the A549 and HaCaT cells after treatment with Cr_2_O_3_ particle A or K_2_Cr_2_O_7_. However, more than a 10-fold higher dose of particle A (88 µg/mL), and up to a 20-fold higher concentration in the case of K_2_Cr_2_O_7_ (1 mM), as well as far shorter treatment times, were applied [10]. Therefore, we assumed that after 24 h of treatment, the initial chromium intermediates responsible for the generation of ROS may have already been vanished due to their instability and short biological half-life [41,42].

Transcriptional alterations were also observed with respect to apoptosis factors. Pronounced repressions were observed for the genes *MAP3K5* and *BTRC*. In case of *MAP3K5,* a downregulation was associated with an increase in cellular stress levels and dysfunction of apoptotic regulatory pathways. Dysregulation can lead to inhibition of programmed cell death, and thus, a shift toward uncontrolled, necrotic cell death mechanisms, some of which result in severe inflammatory responses [43]. However, toxic concentrations of Cr(VI) also resulted in the induction of pro-apoptotic genes, such as *PMAIP*, *PPM1D*, and *TNFRSF10B* in both cell lines. Therefore, the impact on cell death pathways on the transcriptional level appears to be controversial and needs to be further elucidated.

## 5. Conclusions

In conclusion, the results of the present study show that gene expression profiles obtained using high-throughput RT-qPCR provide valuable toxicity profiles for different chromium compounds, reflecting the mode of action of chromium in different oxidation states, especially with respect to the higher toxicity and genotoxicity of Cr(VI) vs. Cr(III). Regarding the Cr_2_O_3_ particles, the cytotoxicity, as well as the gene expression profiles, indicate that the toxicity of the particles resemble either completely (particle 3) or mostly (particle 2) respective profiles obtained for water soluble Cr(III), i.e., showing no or only mild effects, respectively. Therefore, Cr_2_O_3_ nanoparticles or microparticles as such are neither cyto- nor genotoxic. However, this statement holds only for particles not releasing Cr(VI). If the latter is the case, as shown for particle A and—to a much lesser extent—for particle B, observed effects resemble Cr(VI), both qualitatively and even quantitatively. Since the same solubility was observed in distilled water and in artificial lysosomal fluid, Cr(VI) is expected to be released extracellularly, and to be taken up via anion channels, as is known for water soluble Cr(VI). Our findings also have an important impact on the toxicological risk assessment of Cr(III) oxide particles, either in the nano- or in the micro-sized range. Intracellular conversion to Cr(VI) with subsequent reduction, and the subsequent induction of DNA damage, appears to be absent or negligible; also, the same applies to the intracellular release of Cr(III) and the induction of DNA damage. Therefore, the cellular damage depends not on particle uptake, but solely on whether or not Cr(VI) is released from the particles, which needs to be elucidated on a case-by-case basis for risk assessment.

## Data Availability

The data presented in this study are available on request from the first (P.S.) and corresponding author (A.H.) for researchers of academic institutes who meet the criteria for access to the confidential data.

## Supplementary Materials

The following supporting information can be downloaded at: https://www.mdpi.com/article/10.3390/nano12081294/s1, Table S1: Hydrodynamic size of the particles; Table S2: Polydispersity index of the particles; Table S3: Name of coding proteins for the selected genes; Figure S1: complete log_2_ fold gene expression data A549 cells; Figure S2: complete log_2_ fold gene expression data HaCaT cells.
