# Supplementary material for: Impact of Nano- and Micro-Sized Chromium(III) Particles on Cytotoxicity and Gene Expression Profiles Related to Genomic Stability in Human Keratinocytes and Alveolar Epithelial Cells
Source: Nanomaterials (Basel). 2022 Apr 11;12(8):1294. doi: 10.3390/nano12081294 (PMC9029936; doi:10.3390/nano12081294)
Supplement: Supplementary file 1 [file nanomaterials-12-01294-s001.zip › nanomaterials-1643112-supplementary.pdf]

nanomaterials

Supplementary files

Impact of Nano- and Micro-Sized Chromium(III) Particles on Cytotoxicity and Gene Expression Profiles Related to Genomic Stability in Human Keratinocytes and Alveolar Epithelial Cells

Paul Schumacher [1], Franziska Fischer [1], Joachim Sann [2,3], Dirk Walter [4,5] and Andrea Hartwig [1,*]

[1] Department of Food Chemistry and Toxicology, Institute of Applied Biosciences (IAB), Karlsruhe Institute of Technology (KIT), Adenauerring 20a, 76131 Karlsruhe, Germany; paul.schumacher@kit.edu (P.S.); franziska.fischer@kit.edu (F.F.)
[2] Institute of Physical Chemistry, Justus-Liebig-University Giessen, Heinrich-Buff-Ring 17, 35392 Giessen, Germany; joachim.sann@phys.chemie.uni-giessen.de
[3] Center for Materials Research (LaMa/ZfM), Justus-Liebig-University Giessen, Heinrich-Buff-Ring 16, 35392 Giessen, Germany
[4] Institute of Occupational and Social Medicine, Laboratories of Chemistry and Physics, Justus-Liebig-University Giessen, Aulweg 129, 35392 Giessen, Germany; dirk.walter@arbmed.med.uni-giessen.de
[5] Institute of Inorganic and Analytical Chemistry, Justus-Liebig-University Giessen, Heinrich-Buff-Ring 17, 35392 Giessen, Germany
* Correspondence: andrea.hartwig@kit.edu

Supplementary Table S1. Hydrodynamic size of Cr_2O_3 particles in DMEM (10 % FCS) at three different concentrations.

**Particle**	**2 µg/cm²** (10 µg/mL)	**4 µg/cm²** (20 µg/mL)	**10 µg/cm²** (50 µg/mL)
A	177 nm	180 nm	184 nm
B	433 nm	531 nm	575 nm
C	not detectable: particle size too big, rapid sedimentation		

Supplementary Table S2. Polydispersity index (PDI) of Cr_2O_3 particles in DMEM (10 % FCS) at three different concentrations.

**Particle**	**2 µg/cm²** (10 µg/mL)	**4 µg/cm²** (20 µg/mL)	**10 µg/cm²** (50 µg/mL)
A	0.39	0.27	0.22
B	0.68	0.57	0.37
C	not detectable: PDI was too high, particle suspension was not stable		

Supplementary Table S3. Name of coding proteins for the selected genes. Genes marked with * are reference genes.

**Gene**	**Name of coding protein**
*ACTB**	*Β-actin (ACTB)*
APAF1	*apoptotic protease activating factor (APAF1)*
APEX1	*apuirinic-apyrimidinic endonuclease 1 (APEX1/Ref-1)*
ATM	*ataxia telangiectasia mutated (ATM)*
ATR	*ataxia telangiectasia and Rad3-related protein (ATR)*
*B2M**	*beta-2-microglobin (B2M)*
BAX	*bcl2-associated x protein (BAX)*
BCL2L1	*bcl2-like 1 (BCL-XL)*
BRCA1	*breast cancer 1, early onset (BRCA1)*
BRCA2	*breast cancer 2, early onset (BRCA2)*
BTRC	*transducin repeat containing E3 ubiquitin protein ligase, beta (β-TrCP)*
CAT	*catalase (CAT)*
CCND1	*cyclin D1 (CCND1)*
CDKN1A	*cyclin-dependent kinase inhibitor 1A (p21)*
CDKN1B	*cyclin-dependent kinase inhibitor 1B (p27)*
CDKN2B	*cyclin-dependent kinase inhibitor 2B (p15)*
CYP1A1	*cytochrome P450, family 1, subfamily A, polypeptide 1 (CYP1A1)*
DDB1	*damage-specific DNA binding protein 1 (DDB1)*
DDB2	*damage-specific DNA binding protein 2 (DDB2)*
E2F1	*E2F transcription factor 1 (E2F1)*
EGFR	*epidermal growth factor receptor (EGFR)*
EPHX1	*epoxide hydrolase 1, microsomal (xenobiotic) (EPHX1)*
ERCC1	*excision repair cross-complementation group 1 (ERCC1)*
ERCC2	*excision repair cross-complementation group 2 (XPD)*
ERCC4	*excision repair cross-complementation group 4 (XPF)*
ERCC5	*excision repair cross-complementation group 5 (XPG)*
FTH1	*ferritin, heavy polypeptide 1 (FTH1)*
G6PD	*glucose-6-phosphate dehydrogenase (G6PD)*
GADD45A	*growth arrest and DNA-damage-inducible, alpha (GADD45A)*
*GAPDH**	*glyceraldehyde-3-phosphate dehydrogenase (GAPDH)*
GCLC	*glutamate-cysteine ligase, catalytic subunit (GCL)*
GPX1	*glutathione peroxidase 1 (GPX1)*

GPX2	*glutathione peroxidase 2 (GPX2)*
GSR	*glutathione reductase (GSR)*
*GUSB**	*glucuronidase, beta (GUSB)*
HMOX1	*heme oxygenase (decycling) 1 (hMO-1)*
*HPRT1**	*hypoxanthine phosphoribosyltransferase 1 (HPRT1)*
HSPA1A	*heat shock 70kDa protein 1A (HSP70)*
IL8	*interleukin 8 (IL8)*
JUN	*jun proto-oncogene (c-JUN)*
KEAP1	*kelch-like ECH-associated protein 1 (KEAP1)*
LIG1	*ligase I, DNA, ATP-dependent (LIG1)*
LIG3	*ligase III, DNA, ATP-dependent (LIG3)*
MAP3K5	*mitogen-activated protein kinase kinase kinase 5 (MAP3K5/ASK1)*
MDM2	*Mouse double minute 2 homolog proto-oncogene, E3 ubiquitin protein ligase (MDM2)*
MGMT	*O-6-methylguanine-DNA methyltransferase (MGMT)*
MLH1	*mutL homolog 1 (MLH1)*
MSH2	*mutS homolog 2 (MSH2)*
MT1X	*metallothionein 1X (MT1X)*
MT2A	*metallothionein 2A (MT2A)*
MYC	*v-myc avian myelocytomatosis viral oncogene homolog (c-MYC)*
NFE2L2	*nuclear factor, erythroid 2-like 2 (NRF2)*
NFKB1	*nuclear factor of kappa light polypeptide gene enhancer in B-cells 1 (p50/p105)*
NFKB2	*nuclear factor of kappa light polypeptide gene enhancer in B-cells 2 (p49/p100)*
NFKBIA	*nuclear factor of kappa light polypeptide gene enhancer in B-cells inhibitor,alpha (IKBA)*
NQO1	*NAD(P)H dehydrogenase, quinone 1 (NQO1)*
OGG1	*8-oxoguanine DNA glycosylase (hOGG1)*
PARP1	*Poly (ADP-ribose) polymerase 1 (PARP1)*
PCNA	*proliferating cell nuclear antigen (PCNA)*
PLK3	*polo-like kinase 3 (PLK3)*
PMAIP1	*phorbol-12-myristate-13-acetate-induced protein 1 (NOXA)*
POLB	*polymerase (DNA directed), beta (POLB)*
POLD1	*polymerase (DNA directed), delta 1, catalytic subunit (POLD1)*
PPM1D	*protein phosphatase, mg2+/Mn2+ dependent, 1D (PPM1D)*
PRDX1	*peroxiredoxin 1 (PRX1)*
RAD50	*RAD50 homolog (S. cerevisiae) (RAD50)*
RAD51	*RAD51 recombinase (RAD51)*
RRM2B	*ribonucleotide reductase M2B (TP53 inducible) (p53R2)*
SEPP1	*selenoprotein P, plasma, 1 (SEPP1)*
SIRT2	*sirtuin 2 (SIRT2)*
SLC30A1	*solute carrier family 30 (zinc transporter), member 1 (ZnT1)*
SOD1	*superoxide dismutase 1, soluble (SOD1)*
SOD2	*superoxide dismutase 2, mitochondrial (SOD2/MnSOD)*
TFRC	*transferrin receptor (TFR)*
TNFRSF10B	*tumor necrosis factor receptor superfamily, member 10b (DR5)*
TP53	*tumor protein p53 (p53)*
TXN	*thioredoxin (TXN)*
TXNRD1	*thioredoxin reductase 1 (TXNRD)*
VEGFA	*vascular endothelial growth factor A (VEGFA)*
XIAP	*X-linked inhibitor of apoptosis (XIAP)*
XPA	*xeroderma pigmentosum, complementation group A (XPA)*
XPC	*xeroderma pigmentosum, complementation group C (XPC)*
XRCC5	*X-ray repair complementing defective repair in Chinese hamster cells 5 (XRCC5)*

Figure S1.1

A549

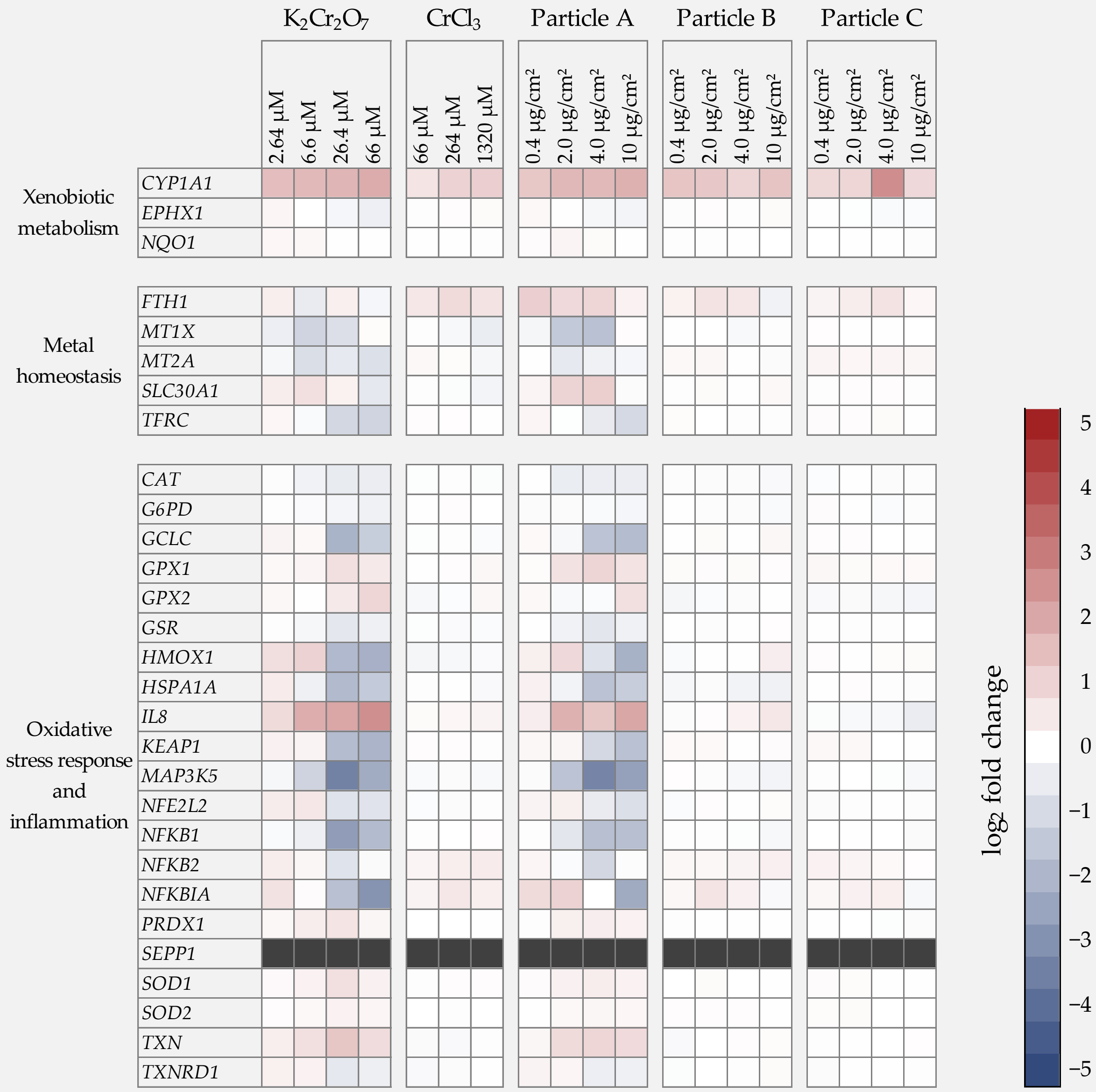

Figure S1.2

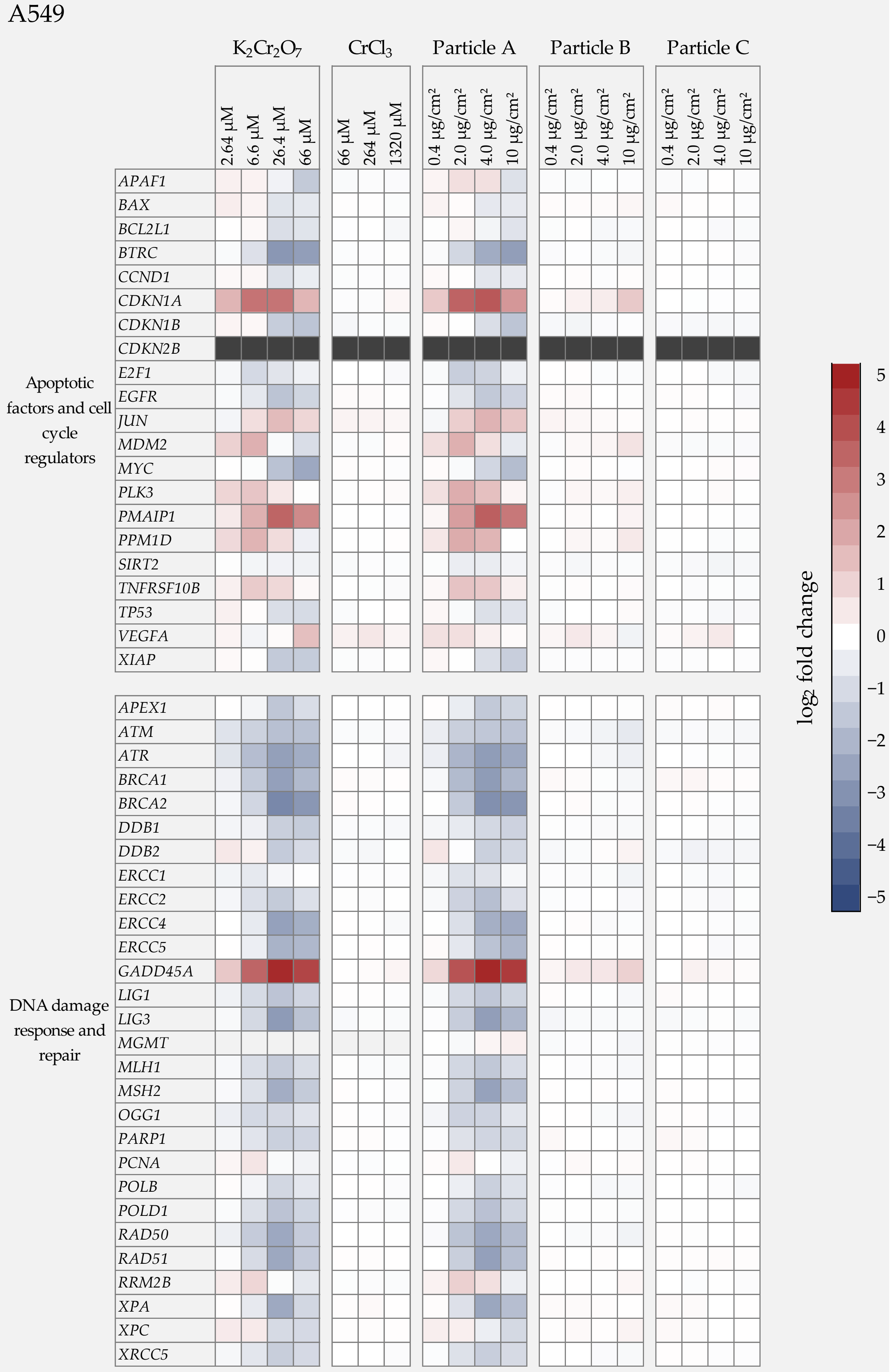
A549
K2Cr2O7
CrCl3
Particle A
Particle B
Particle C
2.64 µM
6.6 µM
26.4 µM
66 µM
66 µM
264 µM
1320 µM
0.4 µg/cm²
2.0 µg/cm²
4.0 µg/cm²
10 µg/cm²
Apoptotic factors and cell cycle regulators
APAF1
BAX
BCL2L1
BTRC
CCND1
CDKN1A
CDKN1B
CDKN2B
E2F1
EGFR
JUN
MDM2
MYC
PLK3
PMAIP1
PPM1D
SIRT2
TNFRSF10B
TP53
VEGFA
XIAP
DNA damage response and repair
APEX1
ATM
ATR
BRCA1
BRCA2
DDB1
DDB2
ERCC1
ERCC2
ERCC4
ERCC5
GADD45A
LIG1
LIG3
MGMT
MLH1
MSH2
OGG1
PARP1
PCNA
POLB
POLD1
RAD50
RAD51
RRM2B
XPA
XPC
XRCC5
log2 fold change
5
4
3
2
1
0
−1
−2
−3
−4
−5

Figure S2.1

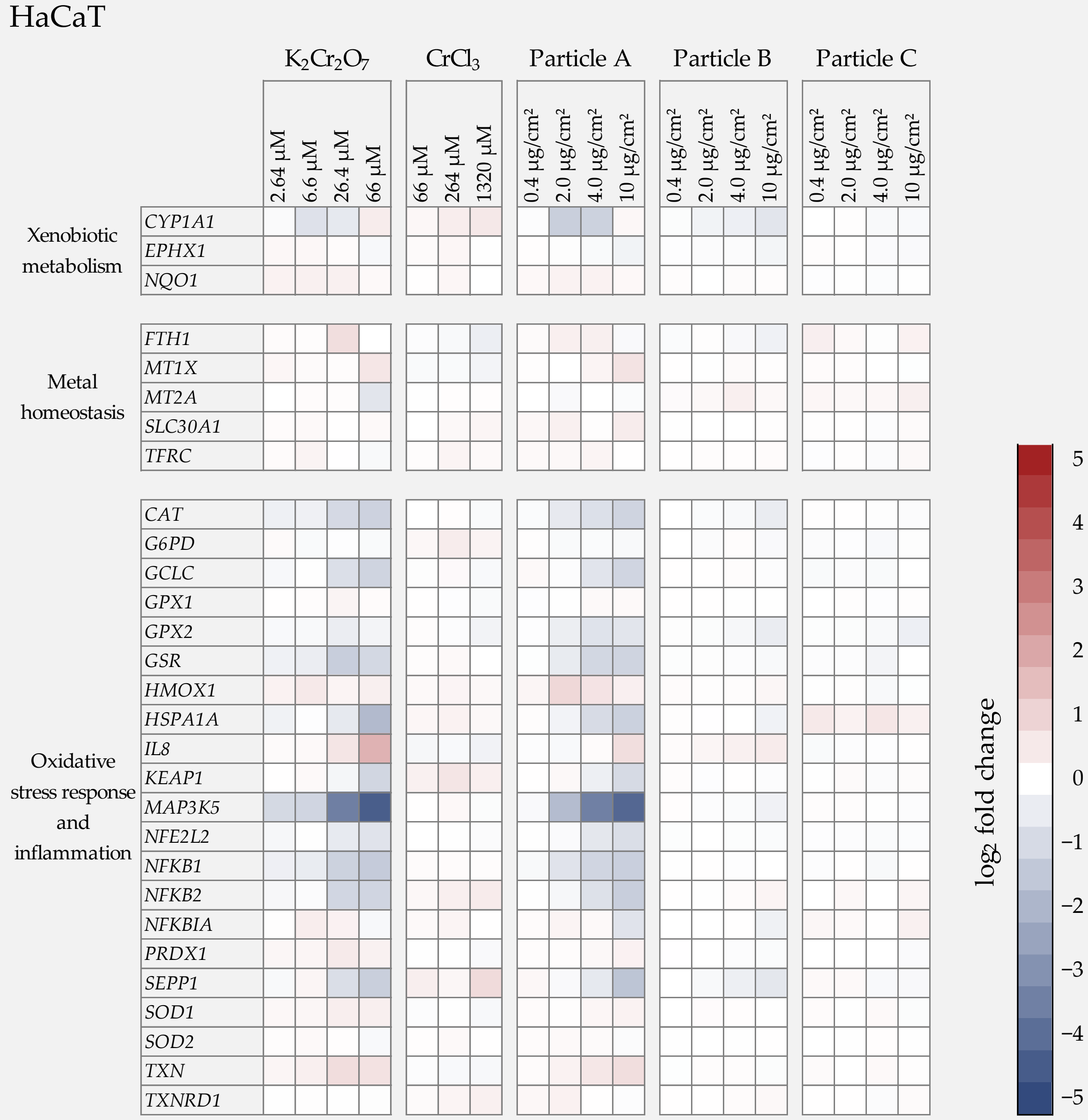
HaCaT
$K_2Cr_2O_7$
$CrCl_3$
Particle A
Particle B
Particle C
2.64 µM
6.6 µM
26.4 µM
66 µM
66 µM
264 µM
1320 µM
0.4 µg/cm²
2.0 µg/cm²
4.0 µg/cm²
10 µg/cm²
Xenobiotic metabolism
CYP1A1
EPHX1
NQO1
Metal homeostasis
FTH1
MT1X
MT2A
SLC30A1
TFRC
Oxidative stress response and inflammation
CAT
G6PD
GCLC
GPX1
GPX2
GSR
HMOX1
HSPA1A
IL8
KEAP1
MAP3K5
NFE2L2
NFKB1
NFKB2
NFKBIA
PRDX1
SEPP1
SOD1
SOD2
TXN
TXNRD1
log_2 fold change
5
4
3
2
1
0
−1
−2
−3
−4
−5

Figure S2.2

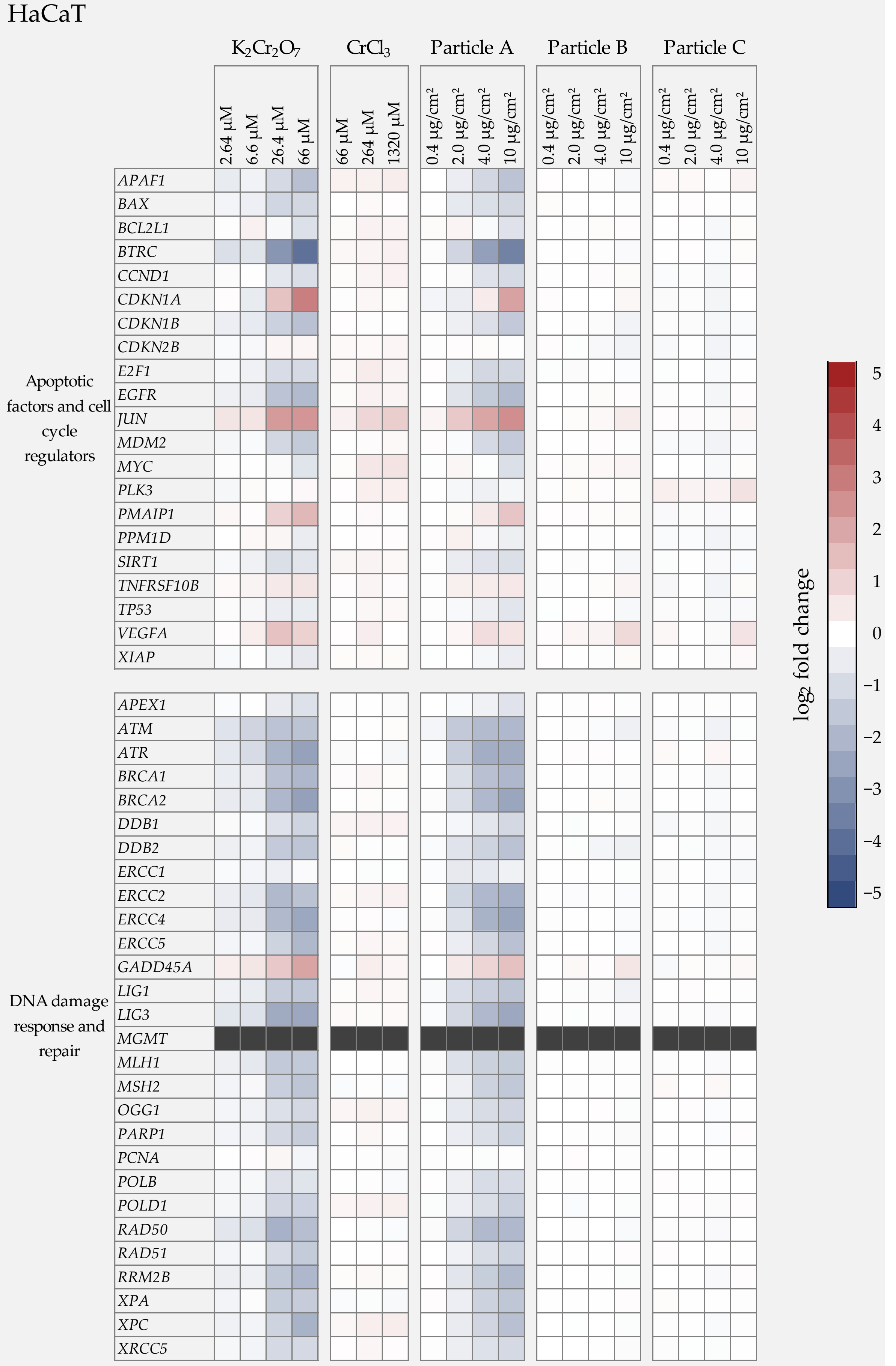
HaCaT
$K_2Cr_2O_7$
$CrCl_3$
Particle A
Particle B
Particle C
2.64 µM
6.6 µM
26.4 µM
66 µM
66 µM
264 µM
1320 µM
0.4 µg/cm²
2.0 µg/cm²
4.0 µg/cm²
10 µg/cm²
Apoptotic factors and cell cycle regulators
APAF1
BAX
BCL2L1
BTRC
CCND1
CDKN1A
CDKN1B
CDKN2B
E2F1
EGFR
JUN
MDM2
MYC
PLK3
PMAIP1
PPM1D
SIRT1
TNFRSF10B
TP53
VEGFA
XIAP
DNA damage response and repair
APEX1
ATM
ATR
BRCA1
BRCA2
DDB1
DDB2
ERCC1
ERCC2
ERCC4
ERCC5
GADD45A
LIG1
LIG3
MGMT
MLH1
MSH2
OGG1
PARP1
PCNA
POLB
POLD1
RAD50
RAD51
RRM2B
XPA
XPC
XRCC5
log_2 fold change
5
4
3
2
1
0
−1
−2
−3
−4
−5
